# Idiopathic macular telangiectasis type 2 and co-existent diabetic retinopathy

**DOI:** 10.1186/s40942-017-0103-x

**Published:** 2017-12-25

**Authors:** Mahima Jhingan, Kerul Marsonia, Dhananjay Shukla, Philip J. Rosenfeld, Jay Chhablani

**Affiliations:** 1 Srimati Kanuri Santhamma Retina Vitreous Center, L.V. Prasad Eye Institute, Kallam Anji Reddy Campus, L V Prasad Marg, Banjara Hills, Hyderabad, Telangana 500 034 India; 2 Ratan Jyoti Netralaya, Gwalior, India; 3 Bascom Palmer Eye Institute, 900 NW 17th St, Miami, FL 33136 USA

**Keywords:** Idiopathic juxtafoveal retinal telangiectasis, Non-proliferative diabetic retinopathy, Proliferative diabetic retinopathy, IMT, Idiopathic macular telangiectasia, Mac Tel2

## Abstract

**Background:**

To study the interaction between idiopathic macular telangiectasis type 2 (MacTel2) and coexistent diabetic retinopathy (DR) during long term follow up.

**Methods:**

A retrospective chart review was done for all eyes with MacTel2 and DR with a minimum 2 years follow up. Eyes with other retinal disorders and poor quality imaging were excluded. Data collected included demographics, presenting visual acuity, systemic evaluation, treatments done, duration of follow up, and final visual outcomes.

**Results:**

Out of 951 patients with MacTel2, 277 patients had diabetes. Out of 277 patients, 44 eyes of 22 patients had MacTel2 coexisting with DR. Twenty-eight eyes of 14 patients were included in this study. All cases of MacTel2 were bilateral with a preponderance of women (71.42%). Mean follow up was 93.07 ± 84.03 months with a mean random blood sugar level of 135.41 ± 45.47 mg% at presentation. Twenty-five (89.28%) eyes presented with mild non-proliferative diabetic retinopathy. Two (7.14%) eyes progressed in their DR staging from baseline. Stage III MacTel2 were noted in 11 (39.28)% eyes at baseline. None of these eyes progressed to stage V during follow up. Mean presenting logMAR BCVA was 0.214 ± 0.227 which dropped to 0.399 ± 0.301 at last visit (p = 0.0005). Diabetic macula edema (DME) was not noted in any eye till last follow up.

**Conclusions:**

12.5% of eyes with MacTel2 in diabetic patients had coexistent DR. MacTel2 led to slowly progressive visual loss irrespective of the presence of DR.

## Background

Idiopathic juxtafoveal retinal telangiectasis or idiopathic macular telangiectasia type 2 (MacTel2) is a condition wherein dilated telangiectatic vessels are present juxtafoveally, usually temporally, and lead to retinal thinning along with photoreceptor damage, and in some instances, subretinal neovascularization secondary to Muller cell degeneration [1, 2]. Gass [1, 2] first described the condition in 1968; since then considerable research has been done to determine its natural history and pathogenesis. Pathogenesis of MacTel2 is still not clear; however, significant association with abnormal glucose tolerance has been reported by Millay et al. [3].

The MacTel research group [4] found a coexistence of diabetes mellitus in 28% and hypertension in 52% in their study, which was suggestive of the fact that the vascular stress in these conditions may add to the pathogenesis of MacTel2. They excluded all individuals who presented with diabetic retinopathy and MacTel2 from their study.

There is very limited literature on natural history of MacTel2 coexistent with diabetic retinopathy. In a case study by Green et al. [5], they noted that the retinal capillary changes in MacTel2 were similar to those observed in the diabetic and pre-diabetic states although the patient had no history of diabetes mellitus. Similar findings were also reported by Diaz-Rodriguez [6] in their study in 2005. Chew et al. [7] compared the Fundus fluorescein angiography (FFA) features of leakage in MacTel2 with diabetic macular edema and explained for the first time the difference in leakage caused due to each one. They underlined the importance of imaging for this differentiation for the very first time. Shukla et al. [8] in a study on the natural history of MacTel2, noted that diabetes was a common association (59%) though retinopathy was initially absent or mild to moderate in 99% patients. However, they did not describe in detail about the natural history of two diseases during the study period.

Reports from the data obtained in the MacTel database [9, 10] were described as abstracts at various meetings and have yet to make it to print as a full paper for our better judgement, but they all focused on the association of diabetes mellitus and MacTel2, besides proving that DR progressed at a slower rate in patients with MacTel. These did not describe the coexistence of DR and MacTel2 as a separate entity besides showing progression.

The aim of this study was to longitudinally study the evolution of MacTel2 (treated and untreated) in the presence of DR; and to assess the effect of presence of MacTel2 on the progression of DR in the largest series so far.

## Methods

A retrospective computer-assisted database search and chart review was carried out on all subjects with a diagnosis of MacTel2 and diabetic retinopathy who presented to LV Prasad Eye Institute, Kallam Anji Reddy Campus, Hyderabad, India, between June 1987 and November 2015 and followed up for a minimum duration of 2 years. Inclusion criteria for our retrospective study included all patients who presented to us between June 1987 and November 2015 and were diagnosed to have MacTel2 and DR at any of their visits clinically with the aid of diagnostics such as fundus fluorescein angiography or optical coherence tomography. Eyes with presence of any other retinal disease or poor quality images were excluded. Eyes with any previous treatment before presentation for either MacTel2 or DR were excluded from the study. Written informed consent had been obtained from all subjects. The institutional review board approved the study and all the procedures adhered to the tenets of the Declaration of Helsinki. The chart review followed the previously set out guidelines, described in earlier publications [11].

Data collected included demographics, corrected distance visual acuity, details of the clinical examination procedure, additional investigations and systemic examination, treatments performed, associated systemic illnesses, duration of follow up in months, and final anatomical and visual outcomes. Visual acuity was measured using the Snellen’s chart and the results were subsequently converted to logMAR notations.

A comprehensive ocular examination was performed in all cases. Ocular investigations included optical coherence tomography (OCT) and fluorescein angiography (FFA), wherever indicated. FFA was performed using FF450plus Fundus Camera with VISUPAC. OCT was performed using Stratus OCT (Carl Zeiss Meditec), RTVue-100 (Optovue) and Cirrus HD-OCT imaging systems (Carl Zeiss Meditec). Systemic investigations included blood sugars or any relevant blood investigations. Diabetes was defined as controlled if random blood sugar was 140 mg/dl or less [12]. A one-step change (documented by fundus photography) in severity of DR was considered a significant change. IJRT 2A or MacTel2 classification and staging was done according to Gass and Blodi classification, [1] using color fundus photographs. All images and data were analysed by single observer (MJ) and in the event of a conflict were reassessed by the second observer (JC), who was responsible for taking the final decision on images.

Treatment modalities employed consisted of observation, laser photocoagulation of non-central diabetic macular edema (DME) in the pre OCT era, intravitreal anti-VEGF agents in cases of CNVM and pan retinal photocoagulation in cases proliferative diabetic retinopathy as per treating ophthalmologist’s discretion. Patients’ follow up was also individualized, as dictated by the disease severity. Repeat FFA was advised in cases of doubts related to progression of DR or development of DME in patients with MacTel.

Statistical analysis was performed using SPSS software (version 16.0; SPSS, Chicago, IL, USA), with special emphasis on the aforementioned information. p values less than 0.05 were considered as statistically significant.

## Results

A total of 951 patients with a diagnosis of MacTel2 in our medical records department database were identified. Out of these, 277 patients were noted to have associated diabetes mellitus.

Out of 951 patients with MacTel2, 277 patients had diabetes. Out of 277 patients, 22 (12.5%) patients had coexistence of diabetic retinopathy and MacTel2 at presentation or during the follow up period, 14 patients among these had a minimum follow up of 2 years and were included in present study. Twenty-eight eyes of 14 subjects fulfilled the criteria to be included in the present study. Ten out of the 14 (71.42%) patients in our study were female; the mean age at presentation was 52.14. ± 5.52 years (range 41–59 years). The pathology was bilateral in all the cases. The median follow up was 68 months with an interquartile range of 79 months (X_U_ 120.25–X_L_ 41.25). The mean duration of diabetes mellitus in the study patients was 11.64 ± 7.056 years. At presentation, the diabetes mellitus was controlled in 11 out of the 14 patients i.e. 78.57% of the study population. Mean random blood sugar at presentation was 135.41 ± 45.47 mg%. At the final visit the history of diabetes mellitus control was specified for only four patients with a mean RBS of 118.2 ± 15.304 mg%.

The other systemic associations, which were present along with diabetes mellitus in our study population are depicted in Table 1. Hypertension was the most common association along with DM with or without other disorders and was found to be present in 50% of the study population.

**Table 1:** Systemic association with idiopathic macular telangiectasis type 2

Systemic association	Number of patients n = 14 (%)
Hypertension alone	4 (28.58%)
Hypertension and coronary artery disease	1 (7.14%)
Hypertension and dyslipidemia	2 (14.28%)
Thyroid disorders alone	1 (7.14%)
None	6 (42.86%)

At presentation, of the 28 study eyes, diabetic retinopathy was noted in all eyes. Most eyes presented with a mild NPDR (25 eyes, 89.28%). Three (10.72%) eyes had PDR at presentation. Two eyes with mild NPDR (7.14%) progressed to PDR during follow up period and required panretinal photocoagulation. The status of diabetic retinopathy among study patients at the first and last visit is as tabulated in Table 2.

**Table 2:** Progression of diabetic retinopathy

Classification of DR	1st visit (%) n = 28	Last visit (%) n = 28
Mild NPDR	25 (89.28%)	23 (82.15%)
Moderate NPDR	0 (0%)	0 (10%)
Severe NPDR	0	0
PDR	3 (10.72%)	5 (17.85%)

At presentation 25 eyes were diagnosed with MacTel2. One eye presented with PDR with subhyaloid haemorrhage, which cleared over the course of the next 6 months and then MacTel2 became apparent. None of the eyes progressed to Stage V [subretinal neovascular membrane (SRNVM)] during follow up; only those patients who presented with stage V MacTel2 were eligible for treatment. The status of MacTel2 at presentation and at last visit is shown as Table 3.

**Table 3:** Progression of idiopathic macular telangiectasis type 2

Stage of IJRT (type II)	1st visit (%) n = 28	Last visit (%) n = 28
Stage I	1 (3.57%)	1 (3.57%)
Stage II	3 (10.71%)	2 (7.14%)
Stage III	11 (39.28%)	7 (25%)
Stage IV	7 (25%)	15 (53.57%)
Stage V	3 (10.72%)	3 (10.72%)
No MacTel2	3* (10.72%)	0

* Three eyes which did not have MacTel2 at baseline, included 2 eyes of one patient in whom early MacTel2 may have been missed and later, the patient presented to us 2 decades later with exudative complications. The third case was a patient with PDR who at presentation had a subhyaloid haemorrhage making assessment of macula and comment of presence of MacTel2 difficult

The largest change noted was the progression of eight eyes (28.57%) with Stage III or less at presentation to Stage IV MacTel2. None of the patients developed a Stage V MacTel2 over follow up. At the same time the number of eyes with Stage IV MacTel2 nearly doubled from seven to fifteen eyes.

During the course of follow up one eye developed a central retinal vein occlusion. Another patient developed bilateral anterior ischemic optic neuropathy during follow up which led to vision loss, however, stage of MacTel2 did not change till last follow up.

Twelve (42.85%) of the eyes underwent treatment for standalone DR [5 eyes (41.67%)] or MacTel2 [7 eyes (58.33%)]. Treatment for MacTel2 included focal laser in six eyes and anti-VEGF injections in three eyes (SRNVM). Mean number of anti-VEGF injections for SRNVM received by patients was 1 for all. One patient underwent additional focal laser for the SRNVM following which he achieved quiescence. Treatment for proliferative diabetic retinopathy (PDR) included panretinal photocoagulation in two eyes and intravitreal bevacizumab in two eyes where vitreous haemorrhage precluded photocoagulation. Patients who underwent focal laser were the patients who had presented earlier on in our case series in 1980s when focal laser was being tried for treatment of MacTel2 [13]. The mean logMAR BCVA for patients who underwent focal laser was 0.204 ± 0.25 (Snellen’s equivalent 20/32) which deteriorated to 0.355 ± 0.24 (Snellen’s equivalent 20/45). These patients complained of drop of vision at the immediate next visit but ultimately recovered visual acuity by the final visit over a follow up of a mean of 109 ± 54.85 months (range 39–170 months).

Two eyes underwent treatment for both DR and MacTel2. They received PRP for PDR and intravitreal anti VEGF’s for Stage V MacTel2. None of the patients in our study developed diabetic macular edema.

The mean logMAR BCVA at presentation was 0.214 ± 0.227 (Snellen’s equivalent 20/32). At the last follow up the mean logMAR BCVA dropped to 0.399 ± 0.301 (20/50 Snellen’s equivalent). This drop in visual acuity was found to be clinically and statistically significant (Wilcoxon signed rank test; p = 0.0005).

Total of 17 eyes (10 patients) had MacTel2 Stage III and less at presentation, 15 of these eyes had mild NPDR at presentation with only two had PDR. Among these 10 patients, coexisting hypertension was seen in five patents (without hypertensive retinopathy) and no other comorbidity in four cases.

No patient progressed to a stage V MacTel2 in our study. Eight of the 18 eyes in five patients (44.44%) progressed in their staging of MacTel2 from their first visit staging of stage III or less to stage IV at the final visit during their mean follow up of 112.61 ± 97.83 months (range 24–228 months). Two of these five patients had an associated comorbidity in the form of hypertension and dyslipidemia. In the same set of patients, the severity of diabetic retinopathy remained unchanged. No clinically significant correlation between systemic comorbidity and progression of diabetic retinopathy was noted but the absence of correlation could not be validated due to small number of cases presenting with other comorbidities.

Among the 25 eyes (13 patients) presenting with mild to moderate NPDR, the most frequently occurring stage of associated MacTel2 was stage III, noted in 10 of 25 eyes (40%); followed by stage IV in 7 of 25 cases (28%). Only two (8%) of these eyes in a single patient showed progression of diabetic retinopathy over the course of our study, progressing to PDR. Eyes showing progression of diabetic retinopathy did not show any change in the staging of IJRT 2A. There was no clinical correlation noted with any other systemic comorbidity in these cases.

All the three eyes (2 patients), who presented with stage V MacTel2 had mild NPDR at the time of presentation, which did not progress. Both these patients had associated systemic hypertension as comorbidity. These patients presented with a mean logMAR BCVA of 0.35 ± 0.30 (approximate Snellen’s equivalent 20/40) which remained more or less stable till the last follow up (mean follow up duration = 35.33 ± 23.714 months), when the mean logMAR BCVA was 0.29 ± 0.36 (approximate Snellen’s equivalent 20/40).

In the 7 patients (14 eyes) who had coexisting hypertension, mean logMAR BCVA at presentation was 0.29 ± 0.20 (Snellen’s equivalent 20/39) which dropped to 0.379 ± 0.27 (Snellen’s equivalent 20/48) at the last visit. (Wilcoxon signed rank test, p = 0.1641). Among these 14 eyes, Stage III or less MacTel2 was noted in eight eyes or 57.14% of the cases, with Stage V seen in three of 14 eyes (21.42%). 3 cases of stage III or less MacTel2 progressed to Stage IV over a mean of 33 ± 12.728 months. Similarly mild NPDR was noted in 11 eyes and PDR in three eyes in this group. None of the eyes with NPDR progressed to PDR in this group.

**Fig. 1:**
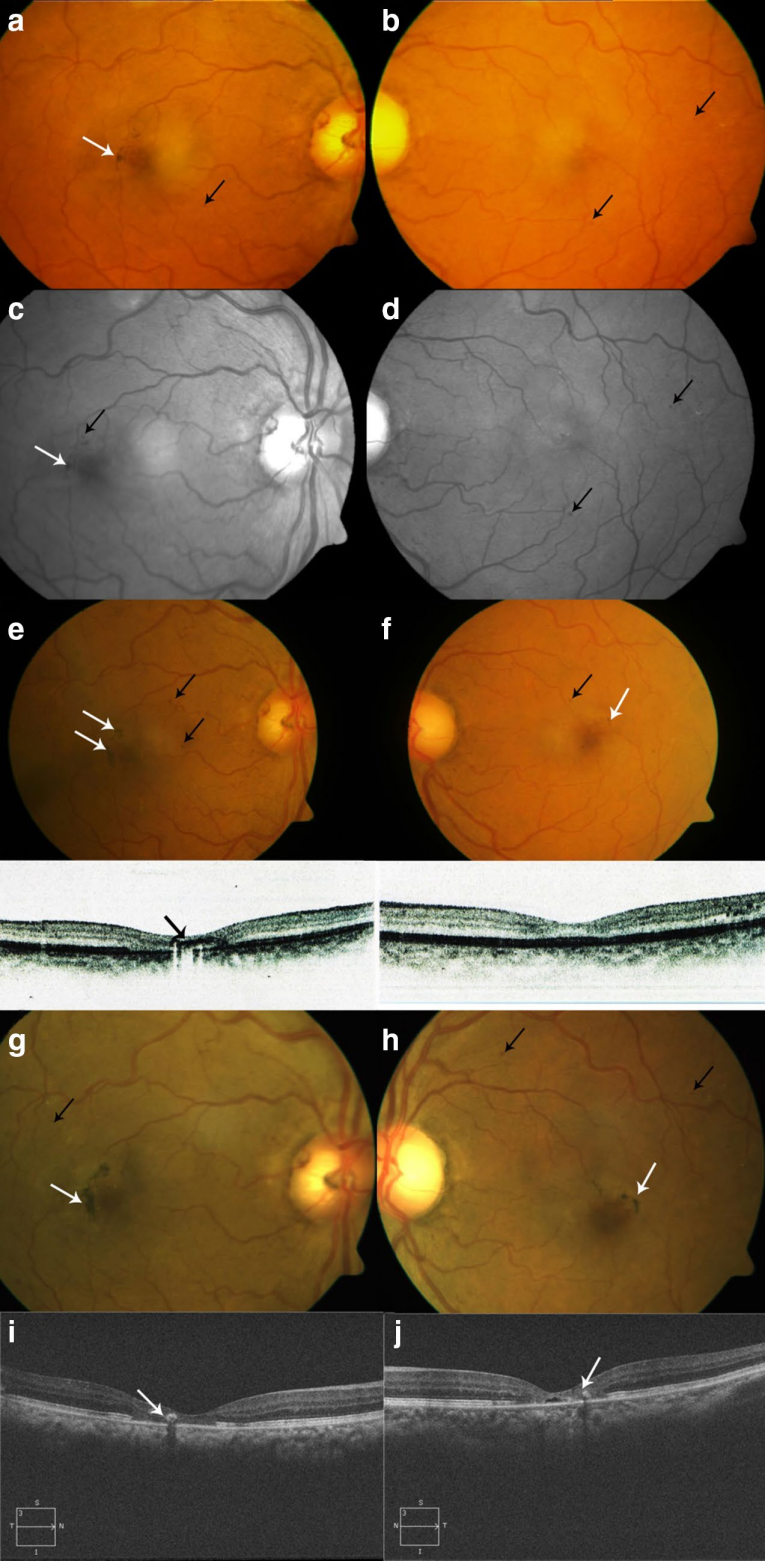
A 56 year old female presented with BCVA of 20/40 (OD) and 20/30 (OS) respectively. Right eye (OD) showed perifoveal greying with RPE hyperplasia plaques temporally (white arrow) and few haemorrhages and microaneurysms (black arrow) (**a**). Left eye (OS) showed perifoveal greying and few intraretinal exudates with few dot hemorrhages (black arrows) (**b**); red free images in OD better delineated the plaques (white arrows) and haemorrhages (black arrows) (**c**). Red free images of OS similarly better delineated the haemorrhages and microaneurysms (black arrows) (**d**). Two years later, BCVA remained stable bilaterally. OD showed an increase in number of plaques (white arrow) whereas haemorrhages and microaneurysms (black arrow) remained similar to the previous visit. SD-OCT showed foveal thinning with disruption of ellipsoid junction with inner retinal hyperreflective lesions (black arrow) corresponding to RPE hyperplasia plaques (**e**). Similarly OS showed the appearance of few plaques (white arrow) not noted earlier with perisstent background haemorrhages (black arrow). SD-OCT Macula showed foveal thinning with disruption of ellipsoid junction (**f**). At her last visit two years later BCVA in both eyes had dropped to 20/160 and 20/60 respectively. OD showed an extensive increase in number and density of plaques (white arrow) along with near similar number of haemorrhages and microaneurysms (black arrow) (**g**). OS showed an increase in number and density of plaques (white arrow) with no significant increase in the number of haemorrhages and microaneurysms (black arrow) (**h**). SD-OCT of OD showed progressive foveal atrophy and further loss of ellipsoid junction besides the hyperreflectivity of the plaques (white arrow) (**i**). SD-OCT of OS also showed an increase in area of defect in ellipsoid junction along with hyperreflective lesions (white arrow) of the plaques in the inner retina (**j**)

Only one patient presented with asymmetric DR in our study with one eye having PDR and the other having mild NPDR. However, MacTel2 staging in both eyes was symmetrical being stage III at presentation. Both the DR and the MacTel2 did not progress till the last follow up. A representative case is shown as Fig. 1.

We also anyalysed the data in the 277 patients of MacTel2 with DM, but who did not have retinopathy along with the remaining 674 patients of MacTel2 without DM, and did a comparative analysis for parameters with our population in all patients who had a follow up of more than 2 years, which was 20 patients of MacTel2 without DM and 23 patients of MacTel 2 with DM without DR. Please find the short comparison in Table 4.

**Table 4:** Comparison of natural history of MacTel2 and DR

	Our study	MacTel2 without DM	MacTel2 wih DM in absence of DR
No. of patients	14	20	23
Mean age (± SD) years	52.14 (± 5.52) years	57.25 (± 8.7) years	56.08 (± 6.6) years
Duration of DM (± SD) years	11.64 (± 7.056) years	NA	9.06 (± 7.35) years
Duration of follow up median (± interquartile rangle) months	68 (± 79) months	71.8 (± 51.75) months	47 (± 17) months
SRNVM formation over follow up (eyes)	None	3	None
Mean BCVA in logMAR at presentation	0.214 ± 0.227 (Snellen’s equivalent 20/32)	0.446 ± 0.5 (Snellen’s equivalent 20/55)	0.345 ± 0.291 (Snellen’s equivalent 20/44)
Mean BCVA in logMAR at final visit	0.399 ± 0.301 (Snellen’s equivalent 20/50)	0.59 (± 0.48) (Snellen’s equivalent 20/77)	0.55 (± 0.42) (Snellen’s equivalent 20/70)

## Discussion

Most studies [13–15] in the past have described a link between MacTel2 and DM on the basis of similar histopathological changes seen in the two and an overall higher incidence of MacTel2 in patients with DM. However, Yannuzzi et al. [16] reported an incidence of 19.2% of the occurrence of DM in patients with MacTel2 which was similar to the rate of occurrence of DM in the general population based studies. National surveys from India shows incidence of DM among metropolitan cities ranging from 6 to 16% [17, 18]. We noted a prevalence of DM of 29.12% in all our study patients of MacTel2 during study period (November 1987 to November 2015), which is higher than the average, mentioned by Yanuzzi et al. Shukla et al. [8] in their study found an incidence of DM in their study population to be 59%, much higher than that noted in any of the previous studies including our study. They did mention a referral hospital induced bias as a possible cause for these values.

Shukla et al. [8] noted the prevalence of mild NPDR in 23 (11%) eyes, moderate NPDR in 24 (12%) eyes, severe NPDR in no eye, and proliferative disease (proliferative diabetic retinopathy) in 2 (1%) eyes, respectively [17]. In our study, we noted a prevalence of DR in 22 patients, i.e. 7.94% of the total diabetics (277 patients) with MacTel2 (951 patients) examined. Two of these patients were not included in our study as they did not meet all our inclusion criteria. This prevalence was far lesser than the study by Shukla et al. This can possibly be explained on the probable tighter glycemic control in our study population.

We found that 44.44% of eyes (8 eyes among 18) of MacTel2 with stage III or less tended to progress over time, with almost no worsening of coexisting diabetic retinopathy over the course of the study. Among these patients showing progression of IJRT 2A, 40% (two patients out of five) had associated hypertension and dyslipidemia. We noted a similar rate of progression of MacTel2 (7 out of 25 eyes) in patients who presented with mild DR (28%) compared with the sample average of 28.57% (8 out of 28 eyes). We also noted that none of the cases with stage IV MacTel2 or less developed an SRNVM during the mean follow up of 98.84 ± 87.242 months. Shukla et al. had similarly found a lower incidence of SRNVM over the course of their study for their diabetic subset (3.45%). The discrepancy of progression between MacTel2 and diabetic retinopathy may be attributed good diabetic control in the study population (which stabilized retinopathy), while IJRT 2A, which is a neurodegenerative process [14] continued to progress.

Similarly, no patient in our study developed DME, showing that the apparent angiographic leakage from telangiectasia does not contribute to the DME [19]. In fact, the neurodegenerative process which leads to foveal atrophy may also reduce the oxygen load of tissues, as thinned out fovea will have lesser metabolic demands. Thus, MacTel2 may have a protective role against DME or progression of DR, a possible hypothesis which requires large scale prospective studies [20].

Systemic vascular shear or stress associated with DM and systemic hypertension are considered as predisposing factors for progression of IJRT 2A [14]. All patients with a stage V MacTel2 in our study had associated systemic hypertension, leading us to speculate that the added vascular stress associated with hypertension could have a possible role in the development of SRNVM due to increased systemic vascular shear in these cases. However, a definite association cannot be made. These patients presented with a mild NPDR, which did not show progression. This supports our hypothesis that in advanced stages of MacTel2, the hypoxic load to the retina is reduced by photoreceptor damage and the chances of progression of DR are subsequently reduced.

We noted that the mean logMAR BCVA at presentation was 0.214 ± 0.227 (Snellen’s equivalent 20/32). At the last follow up the mean logMAR BCVA dropped to 0.399 ± 0.301 (20/50 Snellen’s equivalent). This was a clinically significant drop of more than one Snellen’s lines. Shukla et al. [8] in their study noted a drop of visual acuity from 0.35 ± 0.27 to 0.43 ± 0.28 at the last visit. The drop in visual acuity in our study was higher this could be due to the longer mean duration of follow up in our study (68 months vs. 30 months).

Similar results were noted by Meyer-ter-Vehn et al. [21] who noted a mean visual acuity deteriorated over time-mean visual acuity loss increased from 0.041 logMAR units loss (equivalent to 0.41 lines of visual acuity) after 1 year to 1.2 lines after 3 years, 2.0 lines after 5 years and 2.2 lines after 7 years to finally 4.1 lines after 10 years. Moreover visual acuity decreased from logMAR 0.218 (Snellen’s equivalent 20/33) after 1 year to logMAR 0.25 (Snellen’s equivalent 20/35) after 3 years, logMAR 0.3 (Snellen’s equivalent 20/40) after 5 years to finally logMAR 0.34 (Snellen’s equivalent 20/44) after 10 years. Though the drop in visual acuity was lesser compared to our series, they did not have any coexisting retinal disorders which could worsen the visual prognosis.

Recently, the role of Müller cells in the pathogenesis of a number of retinal disorders including MacTel2 and DR has come to the fore [22]. Neuronal degeneration seen in MacTel2 may be due to oxygen and substrate deprivation during ischemia induced by stressors. To understand the association with diabetes mellitus, Jiang et al. [23] established that one of the key responses of Müller cells to hyperglycemia is an increase in cytokines, specifically IL-1β [24] and TNFα [25]. In fact, Busik et al. [26] suggested that retinal endothelial cells are more responsive to cytokines produced by other retinal cells, compared to high glucose alone. Retinal Müller cells likely respond to the stressor of high glucose with increased GFAP (Glial fibrillary acidic protein) and cytokine levels, thus affecting retinal physiology. Powner et al. [27] compared findings in post-mortem samples of the MacTel2 eye with controls from a healthy patient, a patient with type 2 diabetes and no retinopathy. This difference of expression of GFAP which is a pan glial retinal marker between the two diseases shows that the extent of glial damage is more in diabetic retinopathy, compared to MacTel2 in which the expression of Müller cell specific markers e.g. GS (glutamine synthetase) and CRALBP (cellular retinaldehyde binding protein) are specifically reduced.

Besides the limitation of any retrospective case series, the sample size for our study was small owing to the case selection criteria. Half of the patients presented in the pre-OCT era, therefore, we were unavailable to provide the detailed morphological information about the status at presentation. Though FFA was universally performed at the first presentation for diagnosis and staging, we did not have sequential imaging data for each visit to document the progression of the disease. Despite the limitations, we report the largest case series so far on the coexistence and mutual interaction of MacTel2 and DR and their natural history. Finally, study includes patients over 3 decades, therefore, definition of diabetes has evolved over time and has impact on early diagnosis and staging.

## Conclusion

MacTel2 is a disorder leading to slowly progressive visual loss, which is largely unaffected by the presence of diabetic retinopathy. The severity of diabetic retinopathy at presentation adds to the visual morbidity. Additional systemic associations (e.g. hypertension) are likely to contribute to the progression of MacTel2. While MacTel2 may have a protective role against the development of diabetic macular edema and the progression of the diabetic retinopathy, the results from our investigation suggest that the concurrence of both diseases in the same eye does not have a deleterious effect on the progression of either disease. Further prospective studies are warranted including control group and advanced structural and functional tests to evaluate the effect of both diseases on each other in the same eye.
